# Can improving your aerobic fitness negate the immediate vascular consequences of prolonged sitting?

**DOI:** 10.1113/EP093906

**Published:** 2026-05-28

**Authors:** Molly K. Courish, Myles W. O'Brien

**Affiliations:** ^1^ Faculty of Health Dalhousie University Halifax Nova Scotia Canada; ^2^ Centre de formation médicale du Nouveau‐Brunswick Université de Sherbrooke & Université de Moncton Moncton New Brunswick Canada; ^3^ Department of Medicine Université de Sherbrooke Sherbrooke Québec Canada

**Keywords:** FMD, HIIT, sitting

1

It is well‐accepted that maintaining a high aerobic fitness and limiting time in sedentary postures are crucial components of optimal cardiovascular health. While aerobic training can augment peripheral endothelial function (Shivgulam et al., 2023) in various populations, acute prolonged sitting markedly reduces lower‐limb endothelial function (Paterson et al., 2020). The paper by Kasai et al. (2026) in this issue of *Experimental Physiology* sought to examine what happens when these two opposing movement‐related factors are compared by employing an 8‐week training protocol with pre‐training and post‐training 3‐h sitting protocols. In 22 healthy young adults, Kasai et al. (2026) used a Tabata‐style interval protocol, characterized as eight bouts of 20 s of exercise on a cycle ergometer followed by 10 s of rest. Participants in the intervention group (*n* = 11) underwent three high‐intensity interval sessions a week, supplemented by one body weight session at home. Participants in the control group (*n* = 11) were instructed to maintain their habitual lifestyle, and refrain from initiating any new structured exercise programmes. Aerobic fitness as assessed by ${\dot{V}}_{O_{2}\max}$ was measured via a graded cycle ergometer test before and after the intervention. Before and after the training intervention, participants in both the intervention and the control groups engaged in a 3‐h bout of prolonged sitting. Baseline blood samples (i.e., plasma endothelin‐1 and nitrate concentrations) were obtained. Popliteal artery flow‐mediated dilation (FMD) was assessed via Duplex ultrasonography pre‐sit and each subsequent hour. Resistance vessel function was quantified as the hyperaemic blood velocity area under the curve. Unsurprisingly, Kasai et al. (2026) observed impairments in popliteal FMD and resistance vessel function following 3 h of sitting before any training. Despite the interval training yielding improvements in aerobic fitness (albeit no changes in plasma concentrations of endothelin‐1 and nitrates), 3 h of prolonged sitting similarly impaired popliteal vascular function post‐training. The results observed by Kasai et al. (2026) indicate that an 8‐week interval training programme augmented aerobic fitness but did not attenuate the vascular dysfunction within the popliteal artery during prolonged sitting. These novel findings suggest that the acute sitting‐induced decline in lower‐limb endothelial and resistance vessel function may not be combatted by a Tabata‐style training protocol.

Over the past ∼12 years (Thosar et al., 2015), the acute prolonged sitting model has garnered a lot of attention among researchers employing the FMD technique to study the role of sedentary postures on local blood flow and endothelial function. This is in large part due to the consistently observed marked reductions in leg blood flow (and thus shear stress), which leads to high replicable lower‐limb endothelial dysfunction. To date, lots of work has been conducted testing between‐group differences in the sitting‐induced FMD responses (sex differences, higher vs. lower aerobic fitness, etc.) as well as within‐subject strategies to attenuate dysfunction (heating the limb, activity breaks, etc.). Generally, these within‐subject studies test short‐term manipulations occurring immediately prior to or during the prolonged sitting bout (Paterson et al., 2020). The present study by Kasai et al. (2026) represents an important advancement in the field given its employment of a longitudinal interventional approach in addressing the acute vascular effects of prolonged sitting. Directly manipulating a variable of interest over several weeks, such as aerobic fitness through exercise training, and assessing whether the elevated fitness negates the vascular effects of prolonged sitting is an arduous task that addresses many of the potential biases introduced by between‐subject studies (Liu et al., 2021). This advancement will encourage other prolonged sitting researchers to consider longitudinal interventional models when probing counter‐strategies for vascular effects of prolonged sitting. With time, it is plausible that interventional models focused on diet, activity, pharmacological substances and so forth will begin incorporating before‐and‐after intervention prolonged sitting assessments to determine the optimal strategies to negate the negative health impacts of too much sitting.

It is noteworthy that the authors failed to observe an ability to ‘out‐train’ the negative effects of prolonged sitting (Kasai et al., 2026), providing a much‐needed clarification to whether fitness alters the vascular responses to prolonged sitting (Liu et al., 2021). It is plausible that greater improvements in fitness may be necessary (∼5.3 mL/kg/min following training; Kasai et al., 2026), and an alternative hypothesis for the vasculature may be characterized by the notion of ‘what have you done for me lately?’, in that week‐to‐week or longer modifications may not attenuate the immediate vasoactive consequences of ∼2–3 h of reduced local shear stress. Given that short‐term activity (∼2 min) or standing breaks may prevent sitting‐induced endothelial dysfunction (Paterson et al., 2020), possibly actions that increase local blood flow during the prolonged bout may be one of the few methods of preventing lower‐limb endothelial dysfunction. The advancement of applying interventional models in altering the vascular impacts of prolonged sitting will help yield insights into peripheral vascular control in a posture that comprises a large portion of our daily waking hours. Kasai et al. (2026) should be commended for their intensive study that yields important insights, and the study design advancements it implements represent an important step forward for understanding the interactions between aerobic fitness and seated postures.

## AUTHOR CONTRIBUTIONS

Both authors have read and approved the final version of this manuscript and agree to be accountable for all aspects of the work in ensuring that questions related to the accuracy or integrity of any part of the work are appropriately investigated and resolved. All persons designated as authors qualify for authorship, and all those who qualify for authorship are listed.

## CONFLICT OF INTEREST

M.W.O. is the director of the Sedentary Behaviour Research Network (SBRN), but the material included does not reflect the opinions of SBRN. All other authors have no conflicts of interest to report.

## FUNDING INFORMATION

None.
